# *In Vitro* and Intracellular Activities of Omadacycline against Legionella pneumophila

**DOI:** 10.1128/AAC.01972-19

**Published:** 2020-04-21

**Authors:** Jacques Dubois, Maïtée Dubois, Jean-François Martel

**Affiliations:** a M360 Inc., Sherbrooke, Québec, Canada

**Keywords:** *Legionella pneumophila*, bacterial susceptibility testing, intracellular activities, omadacycline

## Abstract

Omadacycline is an aminomethylcycline antibiotic with *in vitro* activity against pathogens causing community-acquired bacterial pneumonia (CABP). This study investigated the activity of omadacycline against Legionella pneumophila strains isolated between 1995 and 2014 from nosocomial or community-acquired respiratory infections. Omadacycline exhibited extracellular activity similar to comparator antibiotics; intracellular penetrance was found by day 3 of omadacycline exposure. These results support the utility of omadacycline as an effective antibiotic for the treatment of CABP caused by L. pneumophila.

## Abstract (precis)

Omadacycline is an aminomethylcycline antibiotic with *in vitro* activity against pathogens causing community-acquired bacterial pneumonia (CABP). This study investigated the activity of omadacycline against Legionella pneumophila strains isolated between 1995 and 2014 from nosocomial or community-acquired respiratory infections. Omadacycline exhibited extracellular activity similar to comparator antibiotics; intracellular penetrance was found by day 3 of omadacycline exposure.

## INTRODUCTION

Legionnaires’ disease is caused by Legionella pneumophila, a leading cause of atypical community-acquired bacterial pneumonia (CABP; a subset of CAP) (1–3). L. pneumophila is the most common cause of atypical pneumonia in hospitalized patients, second only to Streptococcus pneumoniae in causing severe pneumonia in patients requiring admission to intensive care (4–6). Between 1% and 9% of patients with CABP due to L. pneumophila require hospitalization (7), and mortality rates may reach 10% (2, 6, 8).

L. pneumophila serogroups 1, 4, 5, and 6 are the primary causes of human disease; serogroup 1 is responsible for >80% of reported cases of legionellosis (9). *Legionella* species infect human alveolar monocytes macrophages, and intracellular replication of the bacterium is observed only within monocytes in the phagosomes (10, 11). Antimicrobial agents must, therefore, demonstrate adequate *in vitro* killing activity, intracellular penetration, and *in vivo* activity against L. pneumophila to be effective treatments for Legionnaires’ disease. Typically, macrolide and fluoroquinolone antibiotics are recommended for treating CAP when infection is suspected from atypical bacteria (10). However, because of increased rates of antimicrobial resistance to macrolides and fluoroquinolones (12, 13), alternatives are needed for empirical antibiotic therapy in pneumonia.

Omadacycline is a semisynthetic aminomethylcycline antibiotic derived from tetracycline (14). Omadacycline has *in vitro* activity against a variety of Gram-positive and Gram-negative pathogens, and both *in vitro* and *in vivo* studies demonstrate that omadacycline overcomes the efflux and ribosomal protection mechanisms of tetracycline resistance (15–18). Omadacycline was approved in October 2018 by the U.S. Food and Drug Administration for treatment of CABP and acute skin and skin structure infections and is indicated for the treatment of adult patients with CABP caused by susceptible S. pneumoniae, Staphylococcus aureus, Haemophilus influenzae, Haemophilus parainfluenzae, Klebsiella pneumoniae, and atypical pathogens (Mycoplasma pneumoniae, Chlamydophila pneumoniae, and L. pneumophila) (19). Therefore, the *in vitro* activity of omadacycline against L. pneumophila should be experimentally confirmed.

This study investigated the activity (MIC) of omadacycline and comparators against L. pneumophila isolates from 1995 to 2005 and 2006 to 2014. The minimum extracellular concentration (MIEC) inhibiting intracellular multiplication of L. pneumophila in human monocytes was determined for omadacycline and comparators against L. pneumophila strains.

Antibiotic reference powders were provided by the following groups: Paratek Pharmaceuticals, Inc., King of Prussia, PA (omadacycline, lot number F12-00810 [111483]), Sigma Chemicals, Mississauga, ON (doxycycline, levofloxacin, moxifloxacin, azithromycin, and erythromycin), and Sanofi, Montreal, QC (telithromycin).

Fifty L. pneumophila strains isolated during 1995 to 2005 and 50 strains isolated during 2006 to 2014 (serogroup 1 [*n* = 45] and serogroups 2 to 6 [*n* = 1 per serogroup]) were collected from mostly nosocomial or community-acquired respiratory tract sources. Strains were grown on buffered charcoal yeast extract (BCYE) agar. Five strains of L. pneumophila serogroup 1 were also used to assess intracellular activity. MICs were determined by broth microdilution methodology modified from Clinical and Laboratory Standards Institute (CLSI) guidelines (20, 21).

Against all serogroups of L. pneumophila (*n* = 100), MIC_90_ values for omadacycline (0.06 to 1 mg/liter) were either comparable to, or up to two dilutions lower than, those of azithromycin and erythromycin (Table 1). Against L. pneumophila serogroup 1, the MIC_90_ value of omadacycline (0.25 mg/liter) was lower than the MIC_90_ values of doxycycline, azithromycin, and erythromycin and higher than the MIC_90_ values of telithromycin, levofloxacin, and moxifloxacin (Table 1). Omadacycline was slightly less active against L. pneumophila serogroups 2 to 6 (*n* = 10; MIC range, 0.12 to 1 mg/liter) than against L. pneumophila serogroup 1 (*n* = 54; MIC range, 0.06 to 0.5 mg/liter). Against L. pneumophila serogroups 1 to 6, levofloxacin and moxifloxacin had the lowest MIC_90_ values observed, followed by telithromycin, omadacycline, azithromycin, doxycycline, and erythromycin.

**TABLE 1:** Susceptibility of all tested serogroups of Legionella pneumophila serogroups 1, 2, 3, 4, 5, and 6

L. pneumophila serogroup (no. tested)	Collection dates	Antibiotic	MICs (mg/liter)^*a*^		
			MIC range	MIC_50_	MIC_90_
All (100)	1995–2014	Omadacycline	0.06–1	0.25	0.25
		Doxycycline	0.5–1	1	1
		Telithromycin	0.016–0.12	0.03	0.06
		Azithromycin	0.008–0.5	0.12	0.5
		Erythromycin	0.06–2	0.25	1
		Levofloxacin	≤0.004–0.03	0.016	0.016
		Moxifloxacin	≤0.004–0.06	0.016	0.016
1 (45)	1995–2005	Omadacycline	0.06–0.5	0.25	0.25
		Doxycycline	0.5–1	1	1
		Telithromycin	0.016–0.12	0.03	0.06
		Azithromycin	0.016–0.5	0.12	0.5
		Erythromycin	0.06–2	0.12	1
		Levofloxacin	0.008–0.03	0.016	0.016
		Moxifloxacin	≤0.004–0.06	0.008	0.016
1 (45)	2006–2014	Omadacycline	0.06–0.5	0.25	0.25
		Doxycycline	0.5–1	1	1
		Telithromycin	0.016–0.06	0.03	0.06
		Azithromycin	0.016–0.5	0.12	0.5
		Erythromycin	0.06–2	0.25	1
		Levofloxacin	≤0.004–0.03	0.016	0.016
		Moxifloxacin	≤0.004–0.06	0.008	0.016
2, 3, 4, 5, and 6 (10)	1995–2014	Omadacycline	0.12–1	0.5	1
		Doxycycline	0.5–1	1	1
		Telithromycin	0.016–0.06	0.03	0.06
		Azithromycin	0.008–0.5	0.06	0.5
		Erythromycin	0.12–1	0.25	1
		Levofloxacin	≤0.004–0.008	0.008	0.008
		Moxifloxacin	≤0.004–0.016	0.008	0.008

a MICs determined by broth microdilution in antibiotic concentrations from 0.004 to 128 mg/liter. Standard buffered yeast extract was used against *Legionella* and quality-control strains.

Intracellular activity of omadacycline was determined against five strains of L. pneumophila serogroup 1. The mononuclear cell method (22) was performed using 48-well flat cell culture microplates. RPMI 1640 (with 10% heat-inactivated fetal bovine serum), mononuclear cells (U-937; 1 × 10^6^ to 2 × 10^6^ cells/ml), and *Legionella* inoculum (10^4^ to 10^5^ CFU/ml) were used. After a 1-h exposure in a shaking incubator, 150 μl of infected cultures was maintained without shaking for 7 days at 37°C in 5% CO_2_ and 95% air. After 24 h (day 1), infected cultures were washed three times (300 μl). Antibiotics (150 μl of diluted antibiotic at 1× MIC) were added for a final volume of 300 μl, and cultures were incubated for 2 days. After 72 h (day 3), cultures were washed three times and split into two groups—one with the same antibiotic and one without antibiotic (to observe potential intracellular postantibiotic effect)—for 4 days of incubation. Monocytes in a 20 μl sample taken at time zero and every 24 h until day 7 were diluted by 10-fold dilutions and lysed with distilled water. CFU/ml counts were determined in duplicate using BCYE agar at each time point.

**FIG 1:**
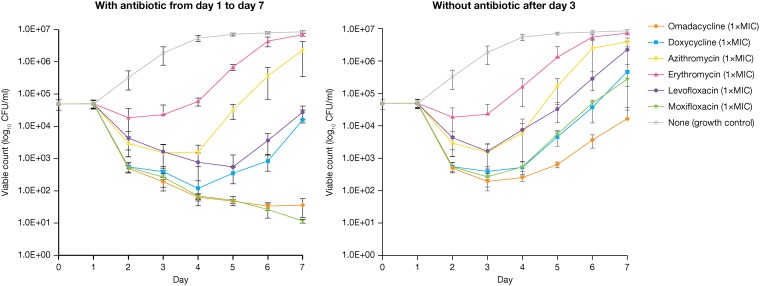
*In vitro* intracellular activity of omadacycline and comparators against Legionella pneumophila serogroup 1 (all five strains: 18, 20, 22, ATCC 33152, and 7) with antibiotic (1× MIC) from day 1 until day 7 of incubation (left) and without antibiotic (1× MIC) after day 3 of incubation (right).

A reduction of 3 log_10_ CFU/ml or 99.9% of L. pneumophila serogroup 1 grown in macrophages was reached only with omadacycline and moxifloxacin after 3 days of antibiotic exposure (Fig. 1). Compared with erythromycin, azithromycin, and levofloxacin, delayed regrowth of intracellular L. pneumophila was observed with omadacycline, moxifloxacin, and doxycycline after drug washout, day 3. A similar reduction and delayed regrowth of intracellular L. pneumophila was obtained at 2× MIC, 8× MIC, and 16× MIC with omadacycline and moxifloxacin (data not shown).

The MIECs of omadacycline and comparators (doxycycline, azithromycin, and moxifloxacin) inhibiting intracellular human monocyte growth (22, 23) were determined for the five strains of L. pneumophila serogroup 1. At days 1 and 3 of exposure, each strain was exposed to antibiotic concentrations of 1, 1/2, 1/4, 1/8, or 1/16 times the MIC required to determine the precise MIEC. Cultures were incubated with antibiotic for 4 days. CFU/ml counts were performed daily in duplicate using BCYE agar. MIEC was defined as the lowest MICs that produced intracellular reductions of ≥1 log_10_ (CFU/ml) of L. pneumophila and was calculated at days 3 and 5 of exposure.

**FIG 2:**
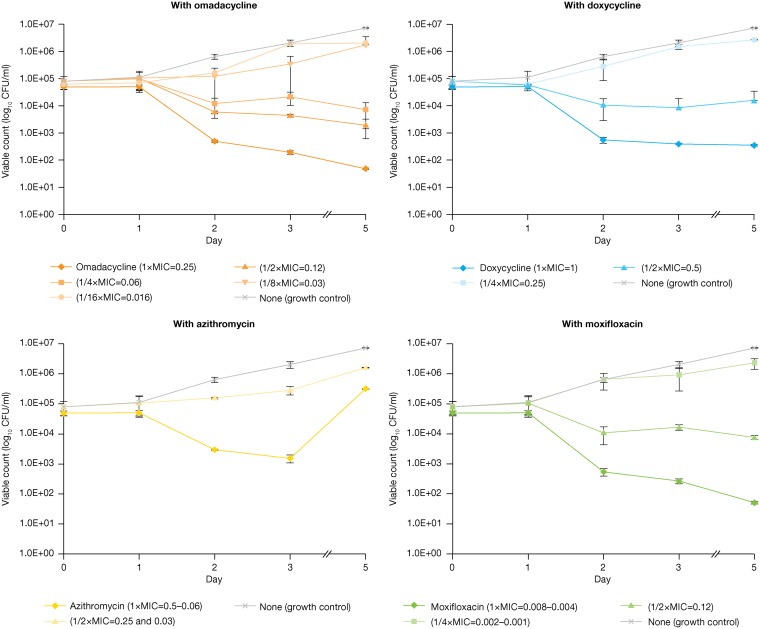
*In vitro* intracellular activity (MIEC) against Legionella pneumophila serogroup 1 (all five strains: 18, 20, 22, ATCC 33152, and 7) with omadacycline (top left), doxycycline (top right), azithromycin (bottom left), and moxifloxacin (bottom right) from day 1 to day 5 of incubation. MIEC, minimum inhibitory extracellular concentration.

Mean reduction of intracellular activity (≥92%) of L. pneumophila growth in macrophages was detected at day 5 of omadacycline exposure, with an MIEC/MIC ratio of 0.24 (1/4× MIC) and MIEC of 0.06 mg/liter (Table 2). At day 3 of omadacycline exposure, an MIEC/MIC ratio of 0.5 (1/2× MIC) and MIEC of 0.12 mg/liter were observed against all tested strains of L. pneumophila (Fig. 2).

**TABLE 2:** MIC, MIEC, and MIEC/MIC ratio of omadacycline and comparators against Legionella pneumophila^*a*^

Antibiotic	MIC^*b*^	MIEC^*b*^,^*c*^; MIEC/MIC ratio by:	
		Day 3 of drug exposure	Day 5 of drug exposure
Omadacycline	0.25	0.12; 0.5	0.06; 0.24
Doxycycline	1	0.5; 0.5	1; 1
Azithromycin	0.5	0.5; 1	>0.5; >1
Moxifloxacin	0.008	0.004; 0.5	0.004; 0.5

a Five strains were tested (18, 20, 22, ATCC 33152, and 7).

b Geometric mean value (mg/liter) for MIC and MIEC.

c MIEC, minimum inhibitory extracellular concentration.

Combining the observed MIEC values at day 5 with the observed mean epithelial lining fluid (ELF) the area under the concentration-time curve from 0 to 24 h (AUC_0–24_) value (17.23 mg · h/liter) and the observed mean alveolar cell (AC) AUC_0–24_ value (302.42 mg · h/l) (24), the estimated AUC_0–24_/MIEC ratio in ELF and AC would be ∼143 and ∼2,520 for tested strains of L. pneumophila, respectively. These important intracellular findings suggest an achievable level of omadacycline at the infection site and support the potency and clinical efficacy of omadacycline for the treatment of CABP caused by susceptible strains of L. pneumophila.

Even when MIC results for doxycycline, moxifloxacin, and azithromycin were lower or higher than those for omadacycline, the MIEC/MIC ratio of omadacycline at day 5 (0.24 or 1/4× MIC) was consistently lower than the MIEC/MIC ratio of moxifloxacin (0.5 or 1/2× MIC), doxycycline (1 or 1× MIC), and azithromycin (>1 or >1× MIC).

Omadacycline demonstrated potent *in vitro* activity against L. pneumophila serogroups 1 to 6. Based on the MIC_90_ values, omadacycline was 4-fold more potent by weight than doxycycline and erythromycin; omadacycline MIC_90_ values were 2-fold lower by weight than that of azithromycin. Omadacycline was 10-fold less potent by weight than telithromycin and fluoroquinolones tested. Noteworthy was the activity of omadacycline against L. pneumophila serogroup 1, the most common serotype isolated from nosocomial or community-acquired respiratory tract infections. Although L. pneumophila strains were isolated from patients across broad time frames, no change in MIC values was seen for omadacycline or comparators, indicating stable susceptibility across 20 years.

L. pneumophila is isolated as the cause of CAP in ∼2% to 5% of cases, but this incidence increases as much as 2-fold in hospitalized patients and the elderly (7). L. pneumophila is an intracellular pathogen, and understanding the intracellular activity, extracellular activity, and cellular penetration of an antibiotic is necessary to evaluate its potential utility. The current study results indicate that omadacycline demonstrates relative intracellular penetrance against L. pneumophila serogroup 1, comparable to other antibiotics used for CABP treatment. Findings also support those from a phase 3 study of CABP in which omadacycline was comparable to moxifloxacin, with a 87% early clinical success rate among 37 patients for whom L. pneumophila was identified as the causative pathogen (25). Thus, omadacycline may be a potential option for empirical therapy for CABP, particularly when atypical bacteria, especially L. pneumophila, are suspected.
